# Non-ionic surfactant vesicles exert anti-inflammatory effects through inhibition of NFκB

**DOI:** 10.1186/s12950-024-00419-5

**Published:** 2024-11-26

**Authors:** Jonathan McGahon, Stuart Woods, Riccardo D’Elia, Craig W. Roberts

**Affiliations:** 1 Strathclyde Institute of Pharmacy and Biomedical Sciences, University of Strathclyde, 161 Cathedral Street, Glasgow, G4 0RE UK; 2 Chemical Biological and Radiological Division, Dstl, Porton Down, Salisbury, SP4 0JQ UK; 3 School of Health and Life Sciences, University of West Scotland, Stephenson Place, Glasgow, Lanarkshire G72 0LH UK

**Keywords:** Inflammation, Non-ionic surfactant vesicles, Anti-inflammatory, IL-6, Cytokine storm, LPS, Transcriptomics, Metabolomics, NFκB

## Abstract

Inflammation can be an unwanted consequence or cause of debilitating diseases of infectious and non-infectious aetiologies. Current anti-inflammatory medications have several deficiencies including lack of specificity and undesirable side effects. Herein, the potential of non-ionic surfactant vesicles (NISV) comprised of monopalmityol glycerol, dicetyl phosphate and cholesterol) as an anti-inflammatory drug and their mode of action is investigated. NISV were able to inhibit LPS-induced IL-6 from BMD macrophages. The individual components of NISV, monopalmityol glycerol, dicetyl phosphate and cholesterol did not affect LPS induced IL-6 levels, proving that formulation of NISV is essential for their anti-inflammatory effects. Transcriptomic analyses showed NISV mediated down-regulation of transcripts for inflammatory mediators in LPS stimulated macrophages. Notably, NISV downregulate NF-κB transcripts in LPS stimulated macrophages. Measurement of inflammatory mediators by cytometric bead array validated a number of transcriptomic findings as NISV were found to inhibit LPS induced IL-6, IL-12, and multiple chemokines. Further investigation demonstrated that NISV inhibited Poly(I:C) or Pam3csk4 induced inflammatory mediators. This indicates that the effects of NISV are distal to both MyD88 and TRIF signalling. Overall, the data generated highlights the potential of NISV as an anti-inflammatory therapeutic.

**Supplementary Information:**

The online version contains supplementary material available at 10.1186/s12950-024-00419-5.

## Introduction

Inflammation is the immune systems response to physical injury or pathogenic infection. Pro-inflammatory mediators such as cytokines are released from immune cells which drive the immune response. This cascade of mediators is often crucial for the clearing of infection or in triggering a healing response [1], however it can also have undesirable consequences to the host such as in cases of runaway cytokine storm or auto immune conditions which may be chronic and life-long [2]. Sepsis is a life threatening condition caused by an overreaction of the immune system to a toxin produced by an infection, such as *Staphylococcus aureus*, present in the bloodstream, called septicaemia [3]. This illness often begins as another form of localised infection, such as a urinary tract or respiratory infection, which is not properly cleared by the immune response and migrates into the bloodstream and continues propagation [4]. At this point, the infection is likely no longer being effectively controlled by the immune system and requires medical intervention. Despite this, the immune system will continue to produce inflammatory cytokines leading to common side-effects such as fever and chills. Without treatment this will lead to organ dysfunction and soon organ failure. Sepsis mortality rate is dependent on the timing of diagnosis and treatment and if found early the mortality rate is 30%, but 50% when the disease advances to severe sepsis. When septic shock occurs mortality is 80% [5]. The treatment of sepsis has been brought into the spotlight with the SARS-CoV-2 pandemic. COVID-19 infections are largely non-lethal, but when complications occur the body becomes septic, and an immune-over reaction takes place causing severe illness, sometimes referred to as viral sepsis, which can lead to death without treatment [6]. Treatment is usually in the form of antibiotics or antivirals to treat the root cause of the inflammation in combination with anti-inflammatories such as glucocorticoids, non-steroidal anti-inflammatories and biologics including soluble cytokine receptors and neutralising antibodies. These options are limited, not completely effective and are associated with some potential severe side effects [7–9].

Non-ionic surfactant vesicles (niosomes) were developed in 1989 and marketed as a cosmetic. Generically they consist of a non-ionic surfactant with or without cholesterol and dicetyl phosphate. They were since developed as immunological adjuvants and drug delivery vehicles capable of carrying diverse cargoes including antivirals, antibiotics and genetic material [10, 11]. The vesicles themselves also serve as a platform for modification with additional components to allow tissue targeted delivery of the vesicles [12] or administration by oral delivery [13, 14]. The studies described herein use non-ionic surfactant vesicles specifically comprised of monopalmitoyl glycerol, cholesterol and dicetyl phosphate (NISV). These were originally developed as a vaccine adjuvant [15], but have been modified for the delivery of various cargos including antivirals, antibiotics and antibodies. However, counter intuitively and in apparent contradiction to their adjuvant effects, NISV consisting of these components have been noted to have anti-inflammatory properties including the amelioration of LPS-induced TNF-a and IL-6 production by macrophages [16].

The following studies were undertaken to confirm these initial observations and to investigate the attributes of these vesicles that account for their anti-inflammatory effects, and to better understand the extent of these effects through using ‘multi-omics’ and cytometric bead arrays. This work also provides mechanistic insight into how the anti-inflammatory effects are mediated.

## Materials and methods

### NISV production

NISV were made using the melt method originally described in Brewer et al. [15]. For the melt method, monopalmitoyl glycerol, cholesterol, and dicetyl phosphate (Sigma Aldrich, UK) were combined in a 5:4:1 molar ratio and heated to 130 °C. Following the addition of warmed PBS, the formulations was vortexed vigorously for 2 min. Vesicle size and zeta potentials were determined by dynamic light scattering using a Zetasizer Nano-ZS (Malvern).

### Bone marrow derived macrophage culture

Bone marrow derived (BMD) macrophages were cultured by flushing the femurs and tibiae of 6–8-week-old male BALB/c mice with DMEM. Stem cells were then cultured in DMEM supplemented with 20% heat inactivated FCS, 15% L929 conditioned media, 5 mM L-glutamine, 100U/ml penicillin and 100μg/ml streptomycin and incubated at 37 °C 5% CO_2_ for 10 days with additional culture media being added on day 3 and media exchange on day 7. On day 10 BMD macrophages were harvested by washing with ice cold PBS and gentle scrapping using a cell scrapper. Harvested cells were centrifuged at 300 g for 5 min and resuspended in RPMI-1640 supplemented with 10% FCS, 100U/ml penicillin and 100μg/ml streptomycin. Live cells were counted using trypan-blue exclusion and cell concentration adjusted to 1 × 10^6^cells/ml. Cells were plated out and incubated at 37 °C 5% CO_2_ overnight before stimulation.

### alamarBlue™ assay

Following macrophage stimulation alamarBlue™ reagent (AbD Serotec, UK) was added to 10% (v/v). Cells were incubated in the dark at 37 °C 5% CO_2_ for up to 6 h. alamarBlue absorbance was then measured using a Spectramax 190 (Molecular Devices) at OD_570_ and OD_600_ and percentage inhibition determined using the method described in McBride et al. [17].

### IL-6 ELISA

To determine the effect of NISV on macrophage viability and IL-6, cultures were treated with 50 µl of vesicles at different concentrations ranging from 3 mM to 0.012 mM in wells with or without LPS at 3 µg/ml. These were then incubated overnight at 37 °C. Macrophage supernatants were collected for IL-6 ELISA using paired IL-6 capture and IL-6-Biotin antibodies from BioLegend. Absorbances read at 405 nm using a Spectramax 190 plate reader (Molecular Devices). IL-6 concentrations were determined against a standard curve (BioLegend).

### LEGENDplex™ cytometric bead array

Macrophages stimulated with LPS at 3 µg/ml, poly(I:C) at 10 µg/ml and pam3csk4 320 ng/ml and treated with 1.5 mM NISV were taken forward for analysis using the LEGENDplex™ Mouse Macrophage/Microglia bead array panel. Macrophage supernatants were collected following stimulation and cytokine content analysed using the LEGENDplex™ Mouse Macrophage/Microglia bead array kit (BioLegend, UK). Supernatant samples and standards were analysed as per manufactures instructions. Analysis was carried out on an Attune NxT (BVR) flow cytometer (Thermo Fisher Scientific, UK). Data was analysed suing LEGENDplex™ Data Analysis Software Suite.

### Transcriptomics and metabolomics

Cells used in Omics experiments were created in one experiment and treated & stimulated identically. Cells were stimulated with 3 µg/ml LPS and treated with a 1.5 mM NISV preparation.

For transcriptomics, macrophage RNA was extracted for transcriptomic analysis 6 h after stimulation. RNA was extracted using the Qiagen RNAeasy mini kit (Qiagen, UK) as per the manufacture instructions. After extraction the RNA quality and quantity was determined using the Bioanalyser 2100 (Agilent) following the manufactures instructions. RNA was stored at -80 °C until transcriptomic analysis. For transcriptomic analysis, RNA was diluted to 1μg g/20μl in RNAse free water shipped to Eurofins for INVIEW Transcriptome Explore analysis. The library preparation was performed by Eurofins Genomics Europe Sequencing GmbH (Konstanz, Germany) using proprietary methods. Subsequent sequencing was also performed by Eurofins Genomics Europe Sequencing GmbH using Illumina HiSeq4000 instruments in 50 bp single end read mode.

Following sequencing, the data is analysed using the ‘tuxedo suite’ beginning with Bowtie, which aligns the reads to a reference genome, then TopHat, which identifies exon-exon splice junctions in said alignment, and finally by the Cufflinks software’s: Cufflinks, Cuffmerge and Cuffdiff. Cufflinks identifies and quantifies the transcripts, Cuffmerge then merges the reads into full-length transcripts and annotates them and Cuffdiff determines the differential expression and measure the significant differences between different samples [18]

For metabolomics, macrophages were incubated with chloroform: methanol: water (20:60:20) for 1 h at 4 °C. Extractions were transferred to 1.5 ml centrifuge tubes where they were vortexed for 5 min and centrifuged at 15,000 g for 3 min at 4 °C. The mass spectrometry analysis was carried out at Glasgow Polyomics. Hydrophilic interaction liquid chromatography was carried out on a Dionex UltiMate 3000 RSLC system using a ZIC-pHILIC column (150 mm × 4.6 mm, 5 μm column). The column was maintained at 25 °C and samples were eluted with a linear gradient (20 mM ammonium carbonate in water, A and acetonitrile, B) over 26 min at a flow rate of 0.3 ml/min. The injection volume was 10 μl and samples were maintained at 5 °C prior to injection. For the MS analysis, a Thermo Orbitrap QExactive was operated in polarity switching mode and the MS settings were as follows: Resolution 70,000, AGC 1e6, m/z range 70–1050, Sheath gas 40, Auxiliary gas 5, Sweep gas 1, Probe temperature 150 °C, Capillary temperature 320 °C. For positive mode ionisation: source voltage + 3.8 kV, S-Lens RF Level 30.00, SLens Voltage 25.00 (V), Skimmer Voltage 15.00 (V), Inject Flatopole Offset 8.00 (V), Bent Flatapole DC 6.00 (V). For negative mode ionisation: source voltage-3.8 kV. The calibration mass range was extended to cover small metabolites by inclusion of low-mass calibrants with the standard Thermo calmix masses (below m/z 138), butyla-mine (C4H11N1) for positive ion electrospray ionisation (PIESI) mode (m/z74.096426) and COF3 for negative ion electrospray ionisation (NIESI) mode (m/z84.9906726). Following mass spectrometry, the raw data was processed by Glasgow Polyomics to generate.MZxml files. These were then analysed using the Polyomics integrated metabolomics pipeline (PiMP) for metabolite identification against known standards [19]

### Statistical analysis

A variety of statistical analyses have been performed using the program Graphpad PRISM V8.0. Graphs have been constructed using Graphpad PRISM V8.0. Continuous data were analysed by parametric analysis (ANOVA, T tests, GLM) when conditions were met (QQ plots to assess Gaussian distribution and Levene’s/Bartlett tests for unequal variation) or non-parametric tests (Kruskal-Wallis, Mann-Whitney, Moods) where these criteria were not met.

## Results

The size, polydispersity index (PDI) and charge of all vesicles were measured and recorded prior to all individual experiments. NISV had a mean size of 1406 ± 22 nm, and a typical PDI of 0.371 and a zeta potential of -29.5mv.

### NISV inhibit LPS-induced IL-6 production at non-toxic concentrations

**Fig. 1:**
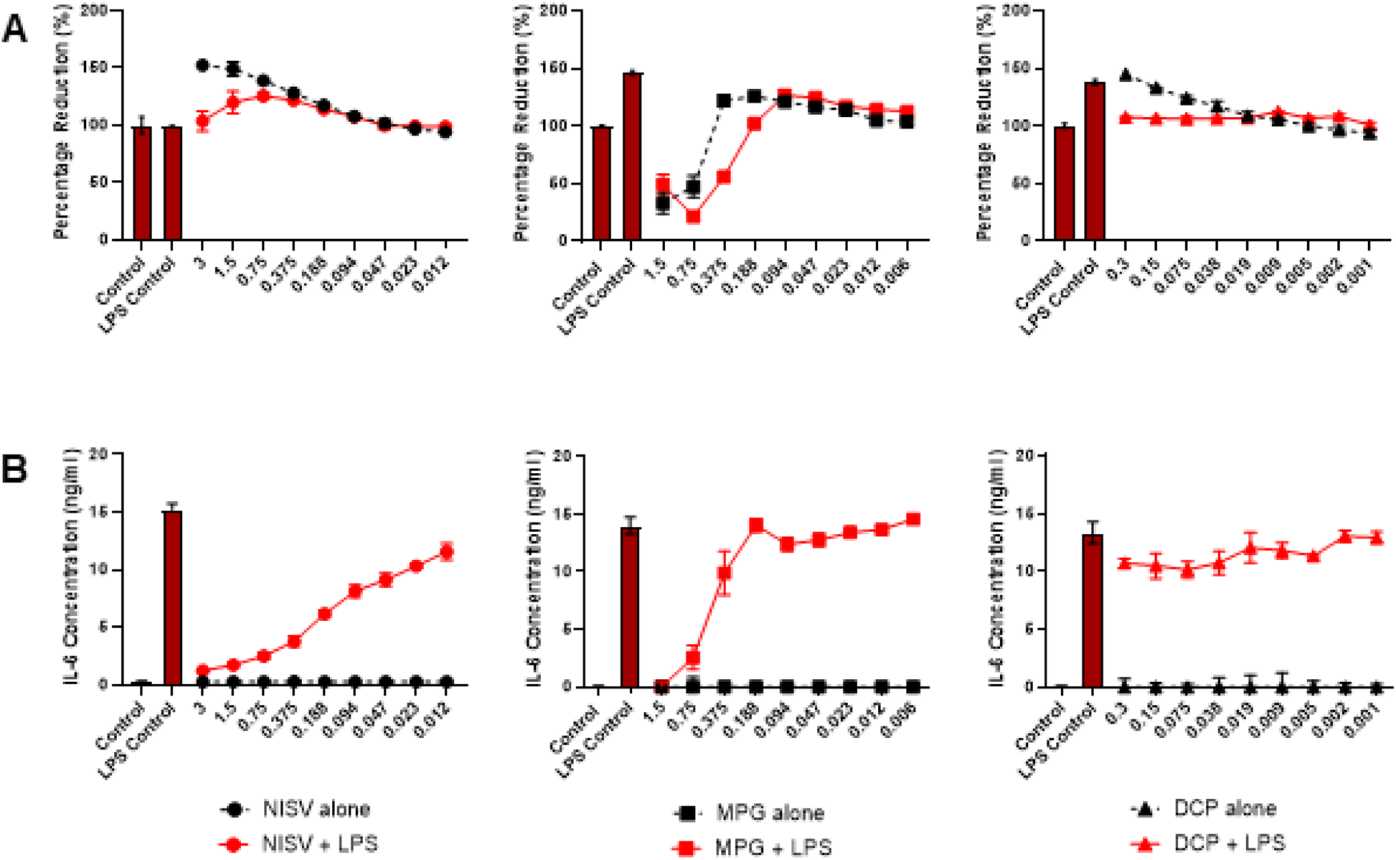
NISV are non-toxic to BMDM and reduce LPS stimulated IL-6 production. 100,000 BMDM were plated per well in triplicate and stimulated with LPS at 3 µg/ml or media, in the controls, followed by treatment with NISV, MPG or DCP at descending concentrations with the controls untreated. **A** shows alamarBlue reduction by BMDM under these conditions and **B** shows IL-6 levels in the supernatants 24 h post-treatment. Statistical analysis was performed on prism 8 using two-way ANOVA and Tukey’s multiple comparisons test. * Indicates statistical significance (*p* < 0.05)

To measure the anti-inflammatory effects of NISV and ensure minimal toxicity an IL-6 ELISA was performed in tandem with an alamarBlue cell viability assay. This assay measures the reduction of resazurin by the cells and is a proxy for general cell metabolism [20]. The results of the alamarBlue assay on Fig. 1A demonstrates NISV at any concentration used have no negative effects on cell viability. At the higher NISV concentrations used the percentage reduction of alamarBlue by BMDMs was increased suggesting that vesicles augmented cellular metabolism.

Figure 1B shows the results of the IL-6 ELISA performed with cell supernatants. Minimal IL-6 was produced by unstimulated cells with or without NISV treatment. The LPS stimulated cells induced > 15 ng/ml IL-6 from BMDM. Treatment of LPS stimulated cells with NISV significantly reduced IL-6 production and followed a dose-dependent pattern. To determine if the immunomodulatory effects of vesicles is dependent on their composition, the individual components of NISV were tested to examine their ability to alter IL-6 production.

Each component was ‘prepared’ using the melt method to more closely mimic the NISV preparation. Figure 1A demonstrates that high concentrations of MPG (0.375-1.5 mM) resulted in a reduction of cell viability. This is likely due to the toxicity of surfactant to cells. Concentrations lower than this were non-toxic to cells and at these non-toxic concentrations there were no obvious changes to IL-6 levels. As expected, IL-6 production was reduced where cells were incubated with toxic concentrations of MPG. Cell viability (Fig. 1A) was not profoundly affected by DCP in the presence or absence of LPS. DCP did not notably affect IL-6 levels in LPS-stimulated macrophages. Cholesterol could not be tested in this manner due to its low solubility and the toxicity of solvents required for solubilisation at required comparable concentrations.

### NISV have minimal impact on macrophage metabolism

**Fig. 2:**
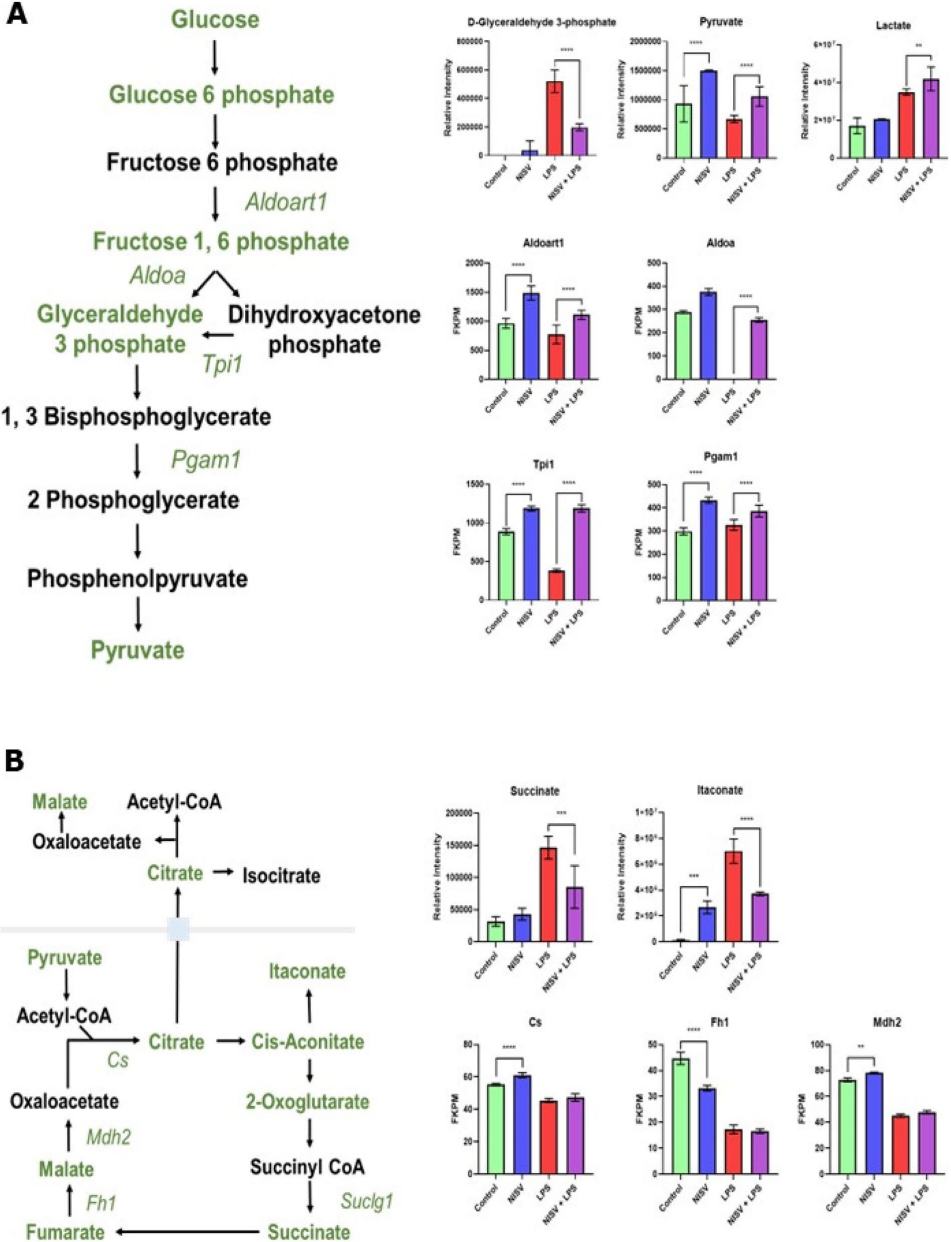
NISV alter the metabolism of BMDM following LPS stimulation. 100,000 BMDM were plated per well as 10 replicates and stimulated with LPS at 3 µg/ml or media, in controls, followed by treatment with NISV. Samples were pooled to make 1,000,000 cells/sample. Cells then underwent metabolite extraction and were sent for metabolomics analysis. Pathway diagrams indicated metabolite detection in green and non-detection in black. **A** shows NISV effects on glycolysis, **B** shows their effects on the TCA cycle. Statistical analysis was performed on prism 8 using two-way ANOVA and Tukey’s multiple comparisons test. * Indicates statistical significance (*p* < 0.05)

The effects of NISV on glycolysis are shown in Fig. 2A. A number of metabolites and transcripts related to glycolysis were significantly affected by treatment with NISV. Levels of the metabolite D-glyceraldehyde 3-phosphate were significantly (*p* < 0.05) upregulated in LPS stimulated BMDM consistent with the ability of LPS to induce the Warburg effect. NISV had little effect on D-glyceraldehyde 3-phosphate levels administered to BMDMs, but ameliorated LPS-induced transcript levels reducing relative intensity from 520853 to 197948. NISV increased pyruvate levels in unstimulated cells (relative intensity 930236 to 1502884) and LPS-stimulated cells (relative intensity 669416 to 1056341). NISV increased lactate levels in LPS-stimulated cells (relative intensity 34926857 to 41934274) but had not affect on unstimulated cells.

Enzyme transcripts were also affected by NISV treatment. NISV increased transcription of Aldoart1 in both stimulated (FKPMs of 775 to 1110), and unstimulated cells (FKPMs 966 to 1487). Aldoa transcription was increased in LPS-stimulated cells by NISV (FKPMs from 0.13 to 255). Triosephosphate isomerase, Tpi1, was increased by NISV in both stimulated (from FKPMs 381 to 1189), and unstimulated cells (from 631 to 1186). Pgam1 shows this same phenotype with NISV increasing transcription, from FKPMs of 298 and 235 to 432 and 385 in unstimulated and stimulated cells respectively. Overall, these data indicate that NISV do not have simplistic effect on glycolysis or indeed LPS-induced Warburg effect.

Figure 2B shows the detected metabolites and transcripts associated with the TCA cycle and the alternate M1 macrophage TCA cycle. NISV reduced succinate and itaconate metabolites significantly in LPS-stimulated cells, reducing the relative intensity of succinate from 146453 to 85161 and itaconate from 7017459 to 3712409. In unstimulated cells NISV increased itaconate levels from relative intensities of 139779 to 2670894. Transcription of citrate synthase, Cs, was significantly upregulated by NISV in unstimulated cells, from an FKPM of 55 to 60. Fh1 transcription was reduced in NISV treated unstimulated cells (FKPM of 44 to 33), but not affected in LPS-stimulated cells. Malate dehydrogenase, Mhd2, transcripts were increased by NISV in unstimulated cells (from FKPM from 72 to 78). Overall, these data indicate that NISV has a limited amelioratory effect on LPS-induced remodelling of the TCA cycle.

### NISV have significant effects on LPS-induced inflammatory transcripts

To further elucidate a potential mechanism of NISV anti-inflammatory effects in LPS-stimulated BMDMs, transcriptomic analysis was performed. Cells were first tested for the characteristic reduction in IL-6 production by NISV in LPS-stimulated cells by ELISA, as well as alamarBlue to confirm no toxicity was detected. PCA analysis demonstrated clear separation between all groups tested and consistency within groups (Supplemental Fig. 1). Approximately 22,000 unique transcripts were detected in each sample. LPS stimulation of BMDM cells significantly altered 11,842 transcripts many of which could be easily identified as immunologically important. In comparison, in NISV stimulated BMDM, 3,830 transcripts were significantly altered (Supplemental Table 1).

**Fig. 3:**
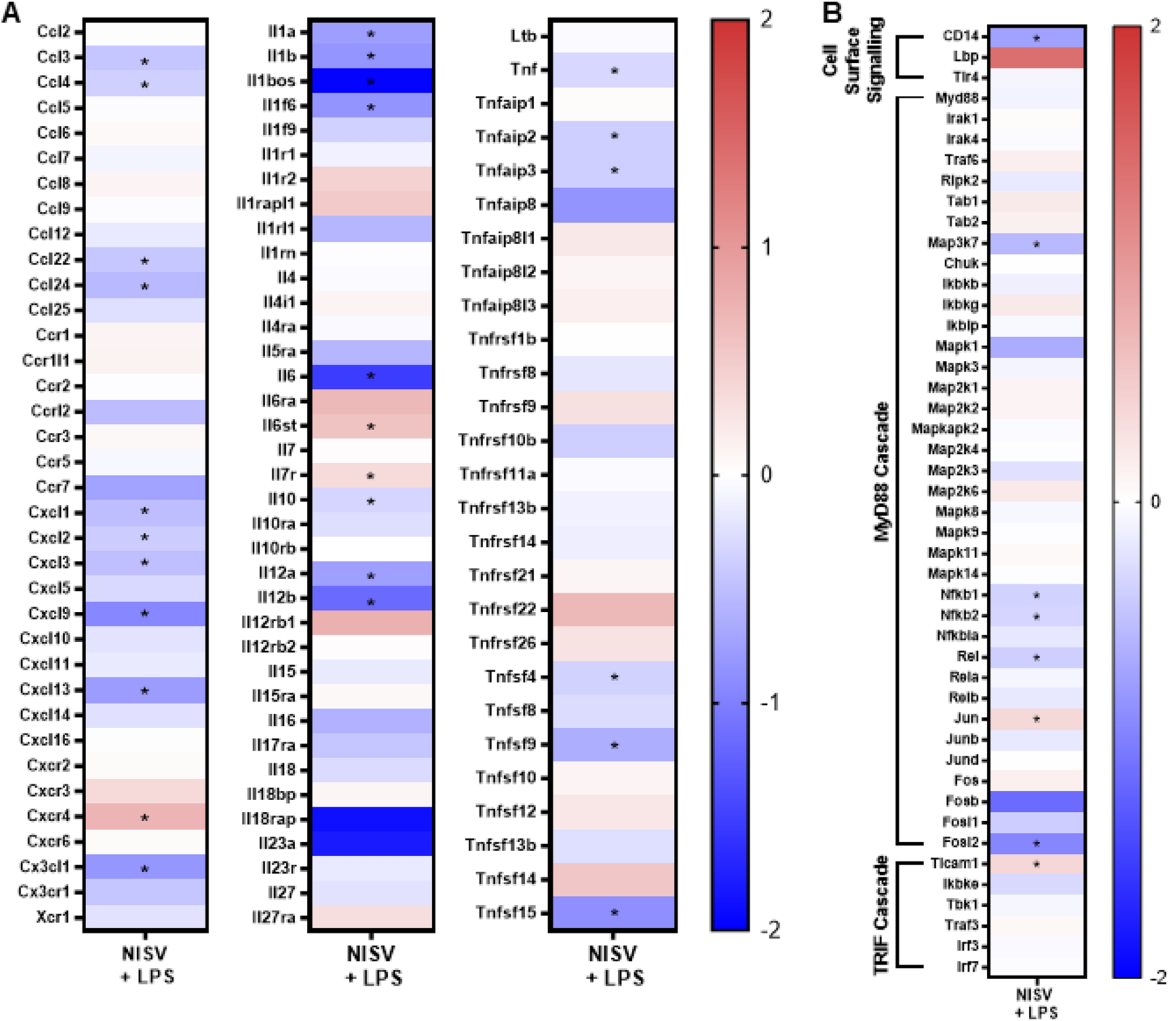
NISV treatment downregulates transcripts induced by LPS stimulation. Transcriptomic analysis was performed on RNA extracted from BMDM stimulated with LPS and treated with 1.5 mM NISV, and BMDM controls untreated with NISV, and unstimulated with LPS. **A** Shows the effects of NISV on the genes responsible for chemokine, interleukin and TNF production in LPS-stimulated BMDM. **B** shows gene expression involved in LPS signal transduction by the TLR4 signalling cascade as they are altered by NISV treatment in LPS-stimulated cells. Heat maps were made using Prism 8. * Indicates significant change. (*p* < 0.05)

NISV were found to significantly alter approximately 626 out of approximately 22,000 transcripts detected in LPS-stimulated cells. NISV treatment of LPS stimulated cells was found to most dramatically affect those transcripts associated by ‘Defence Response’ as determined using the GOrilla analysis tool. Included in this group were chemokines, interleukins, tumour necrosis factor α (TNF-α) and their related genes. It was found that NISV significantly downregulates many genes in these respective groups, including genes of important inflammatory effectors; CCL3, CCL4, CCL22, CCL24, CXCL1, CXCL2, CXCL3, CXCL9, CXCL13, IL-1α & β, IL-6, IL-10, IL-12α & β. In addition, NISV were found to significantly downregulate LPS-induced genes of this family, including Tnf (TNF-α), TNF induced protein expression of Tnfaip2 and Tnfaip3 and superfamily members Tnfsf4, Tnfsf9 and Tnfsf15 (Fig. 3).

As NISV were found to regulate a number of transcripts known to be important in the TLR4 receptor signalling cascade, levels of transcripts involved in affected signalling pathways were examined (Fig. 3A). NISV were found to significantly downregulate Cd14 and Map3k7 (TAK1) and significantly upregulate Ticam1 (TRIF), following LPS stimulation. In signal transduction we see no significant effects in the Map3 kinase cascade, the IKK complex or TRAF-3 signalling. Significant changes are shown in transcription factors expression, NF-κB and AP-1, we show significant downregulation of NF-κB subunits 1, 2 and Rel. AP-1 subunits Jun and Fosl2 are significantly altered by NISV treatment, with Jun being upregulated and Fosl2 significantly downregulated. Many of the genes significantly down-regulated by NISV treatment, in LPS-stimulated BMDMs have their expressions controlled by NF-κB, which is activated upon LPS interaction with TLR4. These findings could indicate that NISV exert their anti-inflammatory effects by down-regulating NF-κB (Fig. 3B).

### NISV inhibit both Trif and MyD88 dependent signalling pathways

**Fig. 4:**
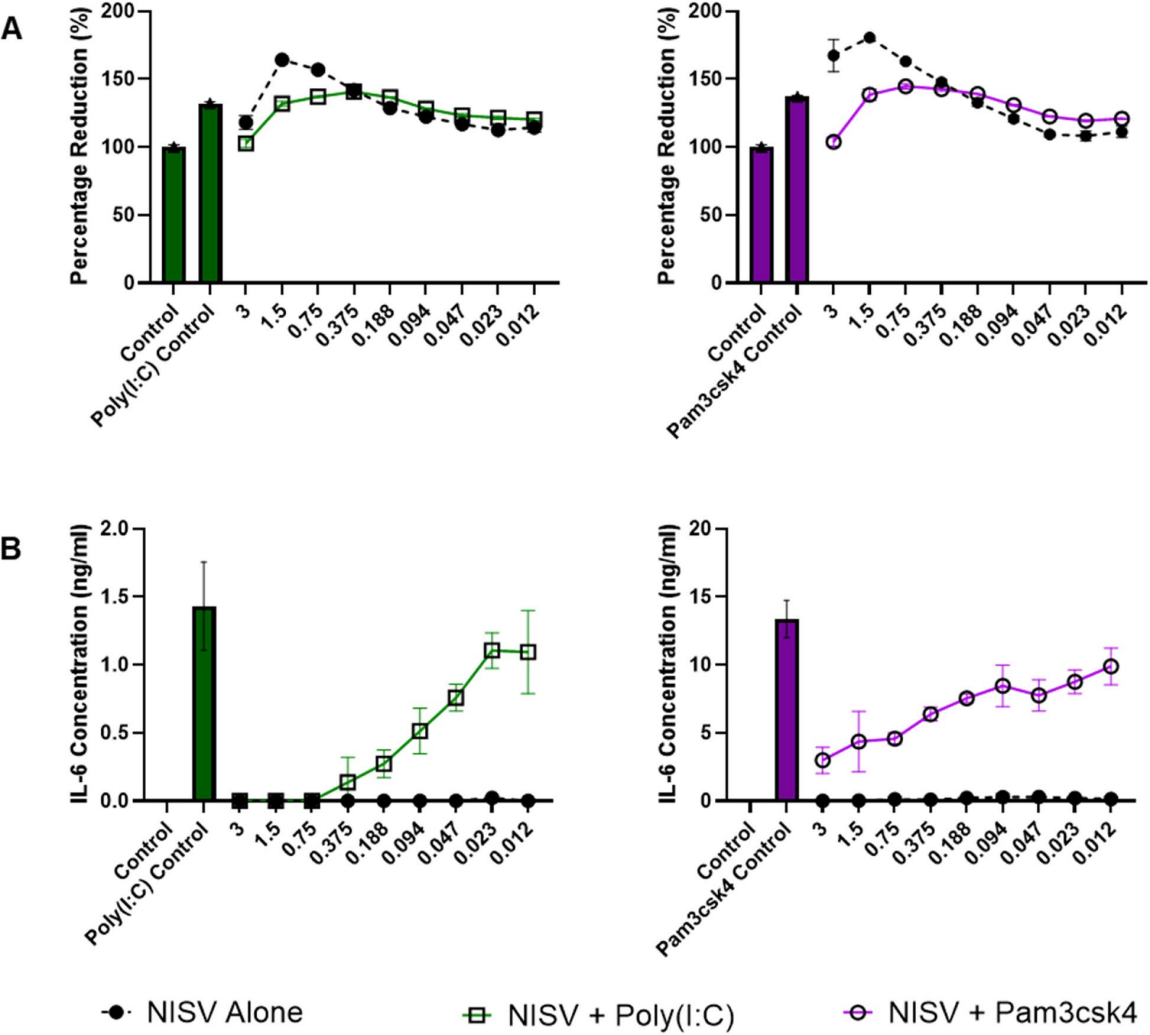
NISV reduce IL-6 response following Poly(IC) or Pam3csk4 stimulation of BMDM. 100,000 BMDM were plated per well in triplicate and stimulated with poly(I:C) at 10 µg/ml, pam3csk4 at 320 ng/ml or media, in the controls, followed by treatment with NISV at descending concentrations with the controls untreated. **A** shows alamarBlue reduction by BMDM under these conditions and **B** shows IL-6 levels in the supernatants 24 h post-treatment

**Fig. 5:**
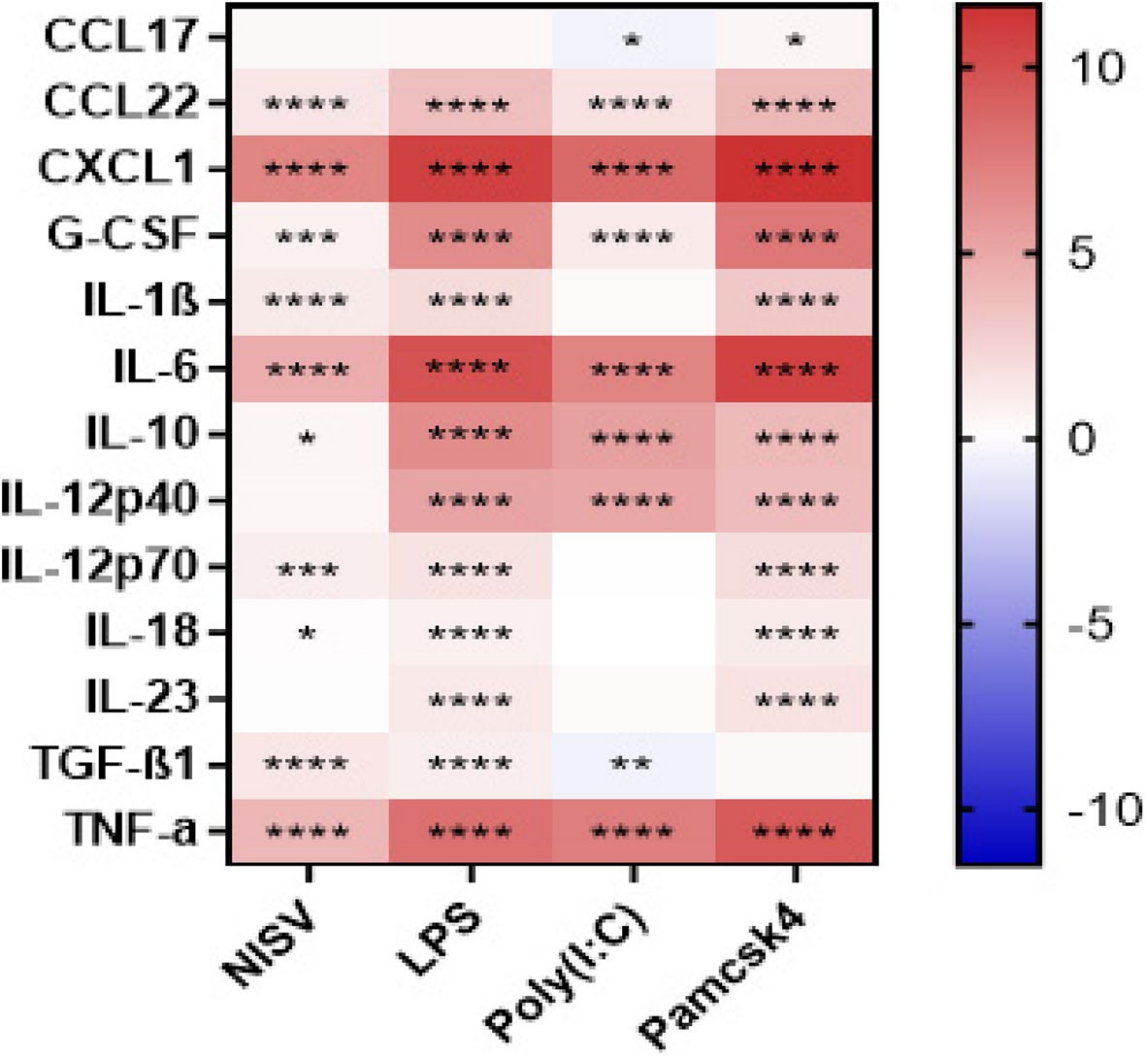
NISV downregulate inflammatory cytokines from BMDM following LPS stimulation. 100,000 BMDM were plated per well in triplicate and stimulated with NISV, LPS, poly(I:C) or pam3csk4 incubated for 24 h. Supernatants were taken immunological markers measured by LEGENDplex™ cytometric bead array using an AttuneNxT flow cytometer. Heat maps were generated showing Log2(fold change) of vesicle and PAMP treatments compared to an unstimulated control. Statistical analysis was performed using Prism 8 where a two-way ANOVA with Tukey’s multiple comparisons

As transcript levels for both myD88 and TRIF were affected by NISV, we investigated the effects of NISV treatment on cells stimulated with Pam3csk4 and Poly(I:C) which initiate signalling through myD88 dependent and TRIFF dependent pathways respectively. NISV inhibited IL-6 production by macrophages stimulated with either of these ligands in dose dependent fashions (Fig. 5). Importantly, none of the concentrations of NISV used had a negative effect on BMDM cells as determined by alamarBlue assay (Fig. 4).

### NISV inhibits inflammatory cytokine production induced by MyD88 and TRIF dependent signalling

**Fig. 6:**
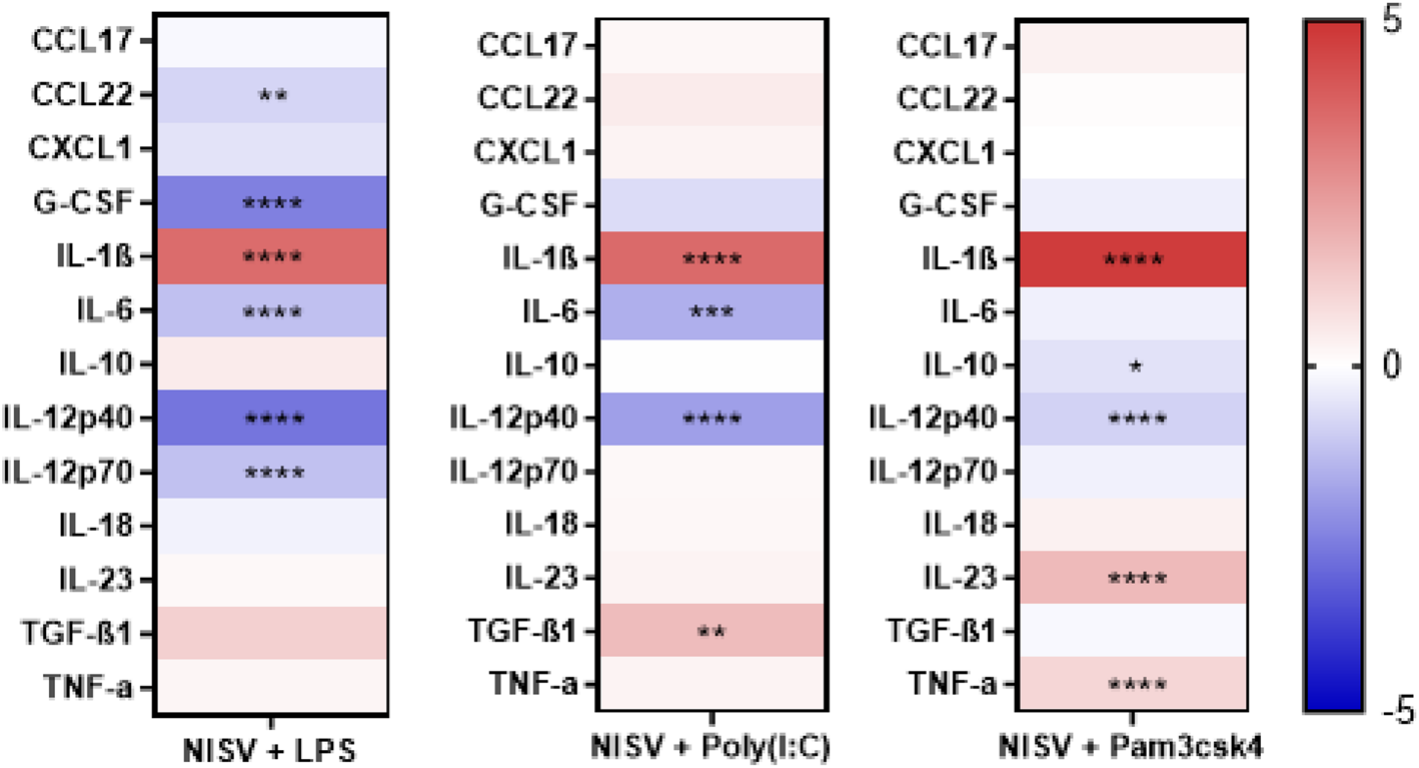
NISV alter inflammatory cytokines from BMDM following LPS, Poly(I;C), or Pam3csk4 stimulation. 100,000 BMDM were plated per well in triplicate and were stimulated using 3 µg/ml LPS, 10 µg/ml poly(I:C) and 320 ng/ml pam3csk4, these were then treated with 1.5 mM NISV. After 24 h supernatants were taken and analysed using a cytometric bead array. Heat map shows the Log2(fold change) of NISVcompared to their respective PAMP control. Statistical analysis was carried out using Prism 8 where a two-way ANOVA and Tukey’s multiple comparisons test was performed

A cytometric bead array was used to investigate NISV effects LPS, Poly(I:C) or Pam3csk induced inflammation and to validate the transcriptomic findings for LPS stimulation. Initial analysis found that each of the TLR stimulants induced several inflammatory mediators by the BMDMs. In addition, NISV alone increased certain mediators to a lesser extent (Fig. 5). However, the anti-inflammatory effects of NISV were evident to varying extents irrespective of TLR stimulant. Thus, NISV were found to significantly decrease (*p* < 0.05) CCL22, G-CSF, IL-6, IL-12p40 and IL-12p70 in LPS-stimulated cells (Fig. 6). They reduced IL-6 and IL-12p40 in Poly(I:C)-stimulated cells and NISV significantly reduced IL-10 and IL-12p40 in Pam3csk4-stimulated BMDM. In all stimulations NISV induced a significant increase in IL-1β. In Poly(I:C) stimulation NISV also increased TGF-β1, and in Pam3csk4-stimulated cells IL-23 and TNF-α production were increased. These data demonstrate that the immunomodulatory abilities of NISV on BMDM are robust, but exhibit subtle, distinct differences depending on the PAMPS used and logically the TLR engaged in macrophages stimulation.

## Discussion

Until the development of biologics that target specific cytokines, the main method for treating inflammation was through steroids. Steroids that target the glucocorticoid receptor are very effective in reducing inflammation, but aside from being immunosuppressive, they have a number of serious side effects. Side effects of corticosteroids include, adrenal insufficiency, reduced ability to control blood sugar levels, osteoporosis, weight gain, cataracts, glaucoma and hypertension. The use of Biologics has been reported to have a range of challenges including immunogenicity and loss of response and the management or prevention of side effects including, auto‐immunity, malignancies, liver function abnormalities, heart failure, and demyelination [21]. Clearly, new strategies for treatment of inflammatory conditions could have potential benefit to many patients suffering from many inflammatory mediated diseases.

NISV consisting of MPG, DCP and cholesterol have been shown to be immunoregulatory and even used as immunological adjuvants. Somewhat counter intuitively to this, they were also reported to be anti-inflammatory [14, 22] The studies described herein revisit these observations, and confirm that NISV modulate the inflammatory response. Moreover, the studies demonstrate that this applies to inflammation induced through myd88-dependent and TRIF dependent signalling as seen through TLR-2, 3 and 4 stimulation. Further, they explore both how the composition of vesicles affects this and by the use of transcriptomics and metabolomics extend knowledge of potential biological mechanisms involved in these effects.

Initial experiments demonstrated that the single components of NISV alone could not replicate the anti-inflammatory effects of these vesicles. MPG reduced alamarBlue metabolism when introduced at high concentrations as a likely result of toxicity due to its surfactant properties. It did not affect IL-6 production at non-toxic concentrations. DCP did not reduce IL-6 production in LPS-stimulated BMDM. Testing cholesterol alone was not feasible due to insolubility. However, the literature would suggest that cholesterol accumulation in macrophages promotes IL-6 and TNF-α production meaning it is unlikely that cholesterol alone would produce the anti-inflammatory effects mediated by NISV [23]. Overall, these results established that formulation of the components of NISV is necessary to mediate the observed anti-inflammatory effects.

The effect of NISV on LPS-stimulation of macrophages was chosen for in depth transcriptomic and metabolomic analyses as it signals through both MyD88 and TRIF dependent pathways. LPS stimulation of macrophages as expected induced a large number of metabolites and transcripts consistent with the literature [24–26] Macrophages treated with NISV had overall relatively modest changes to metabolites and transcripts related to metabolism. However, of particular interest is the ability of NISV to modulate a number of important metabolites and transcripts in LPS-stimulated macrophages. NISV caused important changes in glycolysis in LPS-stimulated BMDM that could be seen as augmenting the Warburg effect with increased pyruvate and lactate production. Commensurate with these observations, a number of transcripts for key enzymes of glycolysis including Aldoart1, Aldoa, Tpi1 and Pgam1 were increased by NISV treatment of LPS-stimulated macrophages. Remodelling of the TCA cycle by LPS as previously reported was confirmed. Thus BMDM stimulated with LPS were found to have increased succinate and itaconate levels. NISV treatment of these cells reduced these two metabolites. Itaconate is known to be produced as a result of inflammation and functions both as an antimicrobial and anti-inflammatory mediator. Its downregulation by NISV in LPS-stimulated cells could be negative feedback due to its function no longer being necessary [27]. Succinate, which is also known to be augmented in macrophages stimulated with LPS and responsible for increased HIF1α stability and downstream cytokine production was downregulated by NISV [28].

In NISV-treated, LPS-stimulated BMDM all significantly affected genes are down-regulated other than CXCR4. Transcripts for the macrophage chemoattractant, CCL3 (MIP-1α), CCL4 (MIP-1β), CXCL2, CXCL3 and CX3CL1, are all significantly down-regulated. This would indicate a potential negative effect on macrophage recruitment caused by NISV treatment [29, 30]. Other downregulated chemokine transcripts are related to leukocyte (CCL22), T Cell (CCL24) and neutrophil (CXCL1) attraction and so NISV also show the potential to reduce recruitment of these immune cells and by proxy the immune response [31, 32]. Most of the chemokines affected are known to be regulated by NFκB (CCL3, CCL4, CCL22, CXCL1, CXCL3 and CX3CL1) and/or AP-1 (CXCL9). NISV were confirmed to downregulate the transcripts for Il1a, Il1b, Il6, Il10, Il12a, Il12b, and tnf all over which are governed by NFκB.

The use of LPS as a stimulant in this study allowed a focused analysis into signalling events in response to TLR4 activation of BMDM and subsequent down-stream events. NISV also reduced CD14 significantly, which could have a negative effect on LPS recognition and so reduce the cells reaction to this stimulant [33–35]. Further down-stream in this signalling cascade, NISV downregulated transcripts for Fosl2 a critical component of the AP-1 transcription factor. Furthermore, there is clear downregulation of transcripts for many NF-κB subunits (Nfkb1, Nfkb2, Rel) in LPS-stimulated BMDM. Together these effects should have profound anti-inflammatory consequences as these transcription factors regulate the expression of many pro-inflammatory systems [36, 37].

To validate these results a number of immune stimulants were used that act through different toll-like receptors and cytokine production examined though a cytometric bead array following NISV treatment. These included LPS as a stimulant of TLR4, poly(I:C) a TLR3 agonist, and pam3csk4, a TLR2 agonist. NISV were shown to mediate dose dependent reduction of IL-6 production in the presence of all three individual PAMPs. The ability of NISV to mediate an anti-inflammatory effect regardless of TLR stimulated, and resulting signalling pathway induced, is consistent with the ability of NISV to alter both MyD88 and TRIF signalling cascades.

It is not easily intuitive to accept that NISV, first designed as an immunological adjuvant have anti-inflammatory properties. However, there is a growing acceptance that vaccine adjuvants can be optimised by carefully modulating their stimulatory properties as excess levels of activation can result in systemic inflammation and reactogenicity. Furthermore, reducing inflammatory mediators produced during vaccination can increase antibody responses [38] Overall the data presented demonstrate the ability of NISV comprised of MPG, DCP and cholesterol to modulate the NFkb pathway and downstream events including the release of inflammatory cytokines. The fact that this effect is independent of TLR and ligand tested (TLR2/PAMcsk2, TLR3/PolyIC or TLR4/LPS) and is therefore not restricted either MyD88 or TRIF dependent pathways, reinforces the broad potential of this strategy for limiting inflammation caused by a broad array of stimuli.

## Supplementary Information

Supplementary Material 1: Supplementary Figure 1. PCA analysis of transcriptomics reveals separation of experimental groups and consistency within groups. 100,000 BMDM were plated per well in triplicate and stimulated with LPS at 3µg/ml or media, in controls, followed by treatment with NISV. PCA analysis was performed using SIMCA 16 do demonstrate separation between groups and within groups. Supplementary Table 1. BMDM transcripts altered by LPS and NISV treatment. Transcriptomic analysis was performed on RNA extracted from BMDM stimulated with LPS and treated with 1.5mM NISV, and BMDM controls untreated with NISV, and unstimulated with LPS. Table shows the total number of transcripts found and how many where significantly altered from the control. Supplementary Figure 2. Volcano Plots showing the Significant Differences found by Treating BMDM with NISV in Unstimulated and LPS-stimulated cells. Transcriptomic analysis was performed on RNA extracted from BMDM stimulated with LPS and treated with 1.5mM vesicle formulations NISV, and the relevant controls, unstimulated cells, and cells treated with NISV alone. NISV caused 3830 significant changes in expression in unstimulated cells and caused 626 significant differences in LPS stimulated cells. Significant difference determined by Cuffdiff (p<0.05).

## Data Availability

Data available from https://pureportal.strath.ac.uk.

## Abbreviations

NISV: Non-ionic surfactant vesicle
MPG: Monopalmityol
DCP: Dicetyl phosphate
LPS: Lipopolysaccharide
Poly(I:C): Polyinosinic:polycytidylic acid
Pam3csk4: Pam3CysSerLys4
S1P: Sphingosine-1-phosphate
