# Supplementary material for: Non-ionic surfactant vesicles exert anti-inflammatory effects through inhibition of NFκB
Source: J Inflamm (Lond). 2024 Nov 26;21:49. doi: 10.1186/s12950-024-00419-5 (PMC11590361; doi:10.1186/s12950-024-00419-5)

**Supplementary Figure 1. PCA analysis of transcriptomics reveals separation of experimental groups and consistency within groups.**

100,000 BMDM were plated per well in triplicate and stimulated with LPS at 3µg/ml or media, in controls, followed by treatment with NISV. PCA analysis was performed using SIMCA 16 do demonstrate separation between groups and within groups.

**Supplementary Table 1. BMDM transcripts altered by LPS and NISV treatment.**

Transcriptomic analysis was performed on RNA extracted from BMDM stimulated with LPS and treated with 1.5mM NISV, and BMDM controls untreated with NISV, and unstimulated with LPS. Table shows the total number of transcripts found and how many where significantly altered from the control.

**Supplementary Figure 2. Volcano Plots showing the Significant Differences found by Treating BMDM with NISV in Unstimulated and LPS-stimulated cells.**

Transcriptomic analysis was performed on RNA extracted from BMDM stimulated with LPS and treated with 1.5mM vesicle formulations NISV, and the relevant controls, unstimulated cells, and cells treated with NISV alone. NISV caused 3830 significant changes in expression in unstimulated cells and caused 626 significant differences in LPS stimulated cells. Significant difference determined by Cuffdiff. (p<0.05).

Supplementary Data

Supplemental Figure 1


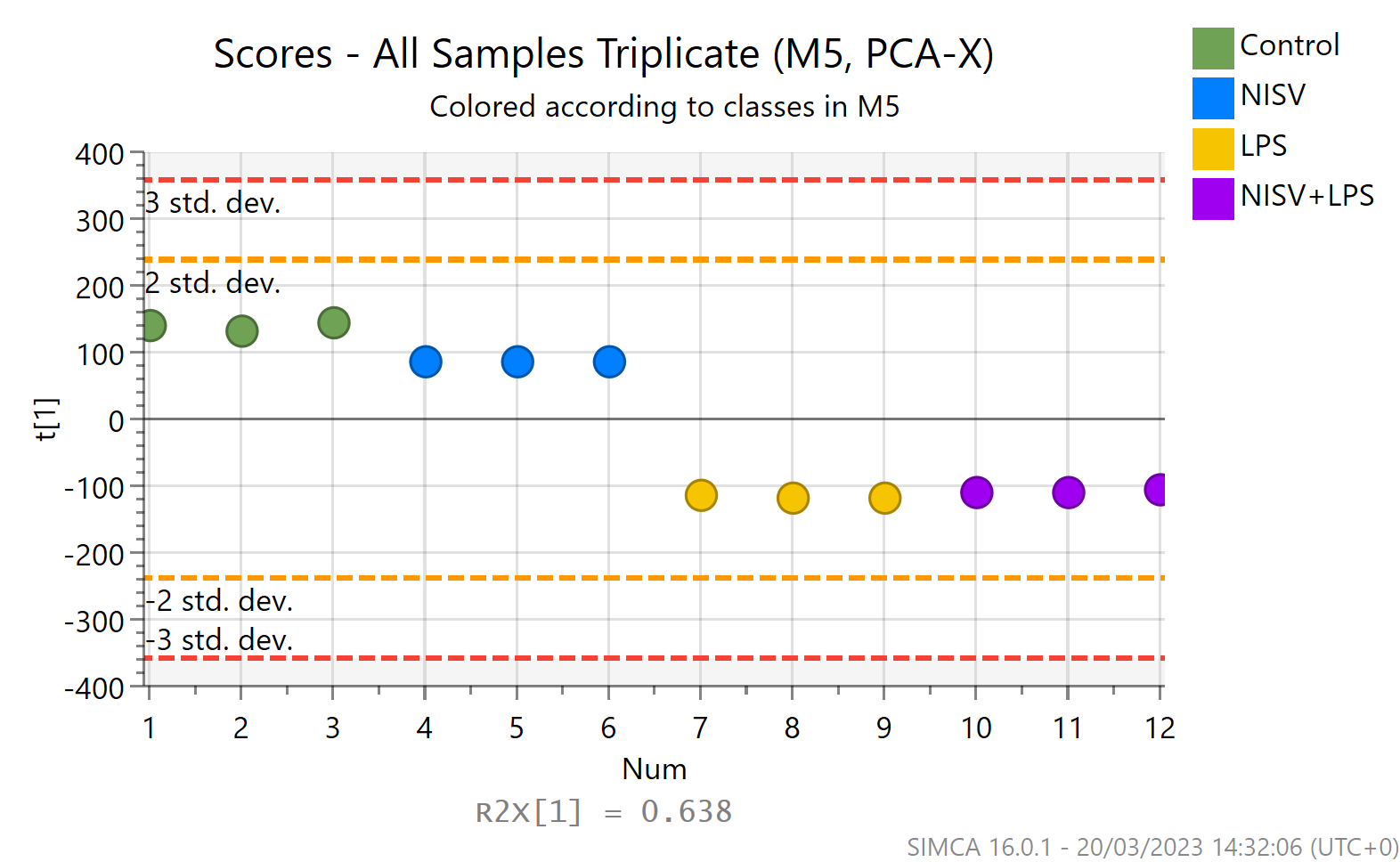


Supplemental Table 1

| Treatment | Number of detected transcripts | Number of Significantly Altered Transcripts |
| --- | --- | --- |
| Control cells | 22,570 | - |
| LPS vs Control | 22,570 | 11,842 |
| NISV vs Control | 22,570 | 3,830 |
| LPS vs NISV LPS | 22,570 | 626 |

Supplemental Figure 2


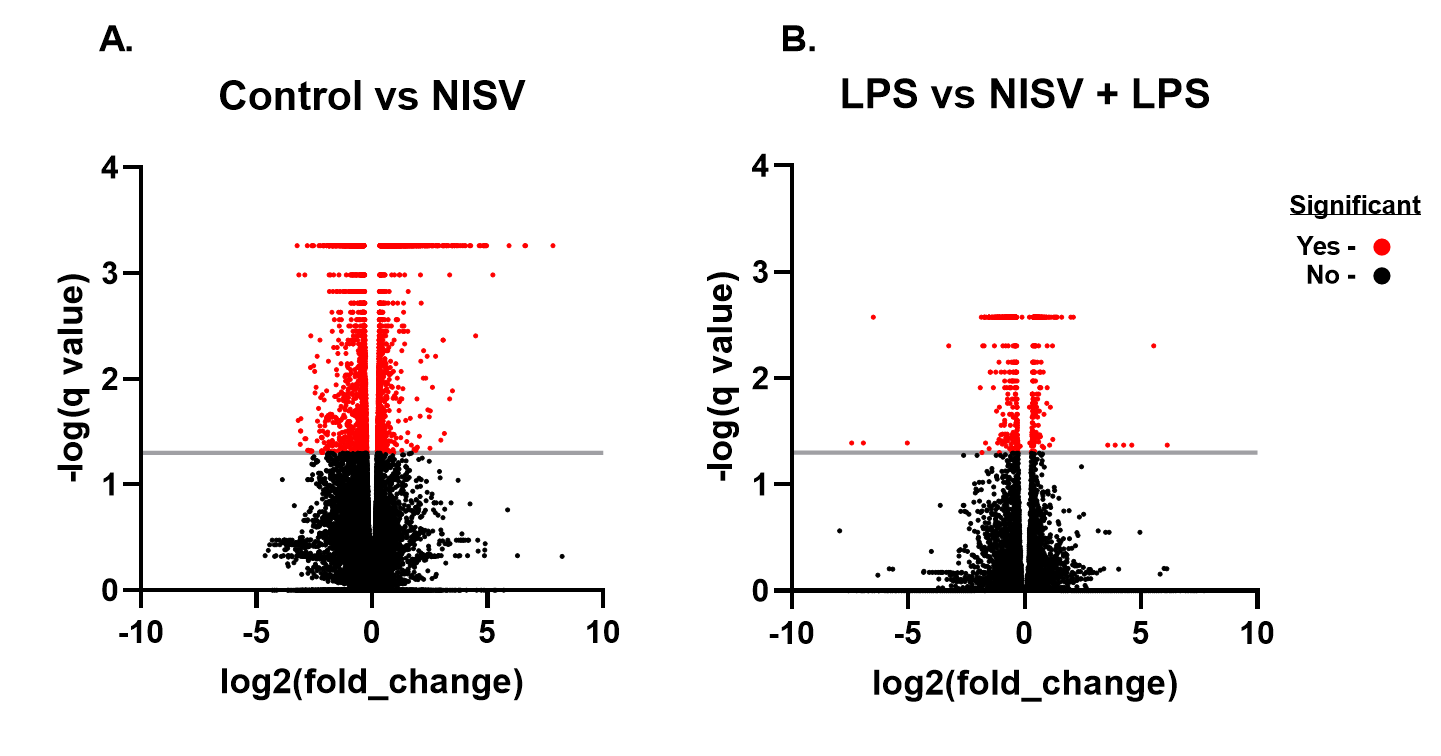

Supplement: Supplementary file 1 — Supplementary Material 1: Supplementary Figure 1. PCA analysis of transcriptomics reveals separation of experimental groups and consistency within groups. 100,000 BMDM were plated per well in triplicate and stimulated with LPS at 3µg/ml or media, in controls, followed by treatment with NISV. PCA analysis was performed using SIMCA 16 do demonstrate separation between groups and within groups. Supplementary Table 1. BMDM transcripts altered by LPS and NISV treatment. Transcriptomic analysis was performed on RNA extracted from BMDM stimulated with LPS and treated with 1.5mM NISV, and BMDM controls untreated with NISV, and unstimulated with LPS. Table shows the total number of transcripts found and how many where significantly altered from the control. Supplementary Figure 2. Volcano Plots showing the Significant Differences found by Treating BMDM with NISV in Unstimulated and LPS-stimulated cells. Transcriptomic analysis was performed on RNA extracted from BMDM stimulated with LPS and treated with 1.5mM vesicle formulations NISV, and the relevant controls, unstimulated cells, and cells treated with NISV alone. NISV caused 3830 significant changes in expression in unstimulated cells and caused 626 significant differences in LPS stimulated cells. Significant difference determined by Cuffdiff (p<0.05). [file 12950_2024_419_MOESM1_ESM.docx]
